# Unidirectional Orbit Determination for Extended Users Based on Navigation Ka-Band Inter-Satellite Links

**DOI:** 10.3390/s25082566

**Published:** 2025-04-18

**Authors:** Yong Shangguan, Hua Zhang, Yong Yu, Wenjin Wang, Bin Liu, Haihan Li, Rong Ma

**Affiliations:** 1 School of Information Science and Engineering, Southeast University, Nanjing 210096, China; wangwj@seu.edu.cn; 2 Beijing Research Institute of Telemetry, China Aerospace Science and Technology Corporation, Beijing 100076, China; yuyong@704.catec.casc (Y.Y.); liubin@704.catec.casc (B.L.); eelihh@outlook.com (H.L.); marong@704.catec.casc (R.M.)

**Keywords:** satellite navigation system, Ka-band inter-satellite links, time–frequency synchronization, emergency communication, orbit determination

## Abstract

Traditional spacecraft orbit determination primarily employs two methodologies: ground station/survey ship-based orbit determination and global navigation satellite system (GNSS)-based orbit determination. The ground tracking measurement system, reliant on multiple tracking stations or ships, presents a less favorable efficiency-to-cost ratio. For high-orbit satellites, GNSS orbit determination is hindered by a limited number of receivable satellites, weak signal strength and suboptimal geometric configurations, thereby failing to meet the demands for the continuous, high-precision orbit measurement of overseas high-orbit satellites. Satellite navigation systems, characterized by global coverage and Ka-band inter-satellite links, offer measurement and communication services to extended users, such as satellites, aircraft, space stations and other spacecraft. With the widespread adoption of navigation satellite systems, particularly in scenarios where ground tracking, telemetry and command (TT&C) stations are out of sight, there is a growing demand among users for Ka-band inter-satellite links for high-precision ranging and orbit determination. This paper introduces an innovative unidirectional orbit-determination technology for extended users, leveraging the navigation Ka-band inter-satellite link. When extended users are constrained by weight and power consumption limitations, preventing the incorporation of high-precision atomic clocks, they utilize their extensive capture capability to conduct distance measurements between navigation satellites. This process involves constructing clock error models, calculating clock error parameters and compensating for these errors, thereby achieving high-precision time–frequency synchronization and bidirectional communication. The technology has enhanced the time and frequency accuracies by three and two orders of magnitude, respectively. Following the establishment of bidirectional communication, unidirectional ranging values are collected daily for one hour. Utilizing these bidirectional ranging values, a mechanical model and state-transfer matrix are established, resulting in orbit-determination calculations with an accuracy of less than 100 m. This approach addresses the challenge of high-precision time–frequency synchronization and orbit determination for users without atomic clocks, utilizing minimal inter-satellite link time slot resources. For the first time in China, extended users can access the navigation inter-satellite link with a minimal allocation of time slot resources, achieving orbit determination at the 100 m level. This advancement significantly enhances the robustness of extended users and provides substantial technical support for various extended users to employ the Ka inter-satellite link for emergency communication and orbit determination.

## 1. Introduction

Traditional spacecraft orbit determination primarily employs two methodologies: ground station/survey ship-based tracking and global navigation satellite system (GNSS) receiver measurements. For medium- and low-orbit spacecraft, the L-band autonomous orbit-determination technology utilizing GNSS navigation receivers has proven effective, offering high accuracy while significantly alleviating the operational burden on ground tracking, telemetry and command (TT&C) networks [1]. However, medium- and high-orbit spacecraft encounter limitations with GNSS navigation signal measurements, including a limited number of receivable satellites, weak signal strength, poor geometric configurations and insufficient robustness [2]. Consequently, orbit measurement and determination for medium- and high-orbit spacecraft predominantly rely on ground tracking stations or ships. This approach, while effective, necessitates multiple tracking stations or ships, resulting in a suboptimal efficiency-to-cost ratio. Additionally, the existing orbit-determination system struggles with tracking arcs for overseas spacecraft, leading to constrained orbit-measurement accuracy. A single tracking station often fails to meet the stringent orbit-determination accuracy requirements for spacecraft.

Satellite navigation systems such as global positioning system (GPS), GLONASS, Galileo and Beidou have established or demonstrated the functionality of Ka-band inter-satellite links. These links enable various extended users (such as satellites, aircraft, space stations and other spacecraft) to perform distance measurements and data transmissions. Particularly when ground TT&C stations are out of sight, high-precision orbit determination can be achieved through the Ka inter-satellite link [3]. While numerous studies have explored orbit determination via navigation inter-satellite links, the majority focus on applications within navigation constellations, with limited research on extended user orbit determination [4,5]. For instance, the study in [6], “Research on the Beidou Clock Difference Observation and Strategy of Autonomous Orbit Determination under the Condition of Inter-Satellite Link”, does not address the challenge of non-high-precision time–frequency extended users achieving time–frequency synchronization, and it only outlines strategies for bidirectional orbit determination between navigation satellites. The paper [7], “The Preliminary Result and Analysis for BD Orbit Determination with Inter-satellite Link Data”, introduces methods for independent orbit determination using Ka inter-satellite links and joint orbit determination combining inter-satellite Ka measurements with L-band data, demonstrating significant improvements in navigation-measurement orbit determination and time synchronization accuracy for the L-band. The papers [8,9] describe methods requiring extended users to use bidirectional ranging values for orbit determination, overlooking the practical limitation that in-orbit extended users can only obtain unidirectional ranging values. These studies also demand frequent observation arcs and substantial time slot resources within the navigation inter-satellite link, which are impractical for large-scale user time slot resource applications.

This paper proposes a unidirectional orbit-determination method based on the navigation Ka inter-satellite link for extended users without high-precision time–frequency references. Given the limited time slot resources of the navigation inter-satellite link [10,11], non-atomic clock users collect unidirectional range data according to time slot planning. The clock difference value is deduced and compensated using a clock difference model, achieving high-precision time and frequency synchronization along with bidirectional communication. Because navigation satellites cannot return ranging values to extended users, the latter can only collect unidirectional ranging values post-launch. Extended users utilize 3–4 inter-satellite link time slots per minute, establishing a link for one hour daily. By constructing a dynamic model and state transition matrix and using the range results from two consecutive days for a sliding solution, orbit-determination accuracy better than 100 m is achieved. This technology addresses the challenge of high-precision time and frequency synchronization and orbit determination for non-atomic clock users using minimal inter-satellite link resources. For the first time in China, extended users can access the navigation satellite inter-satellite link with a small allocation of time slots and achieve 100-m-level orbit determination, significantly enhancing the robustness of extended users and providing robust technical support for various extended users to utilize the Ka inter-satellite link for emergency telemetry and orbit determination.

## 2. The Basic Process of Navigation Inter-Satellite Link Access and Orbit Determination

**Figure 1:**
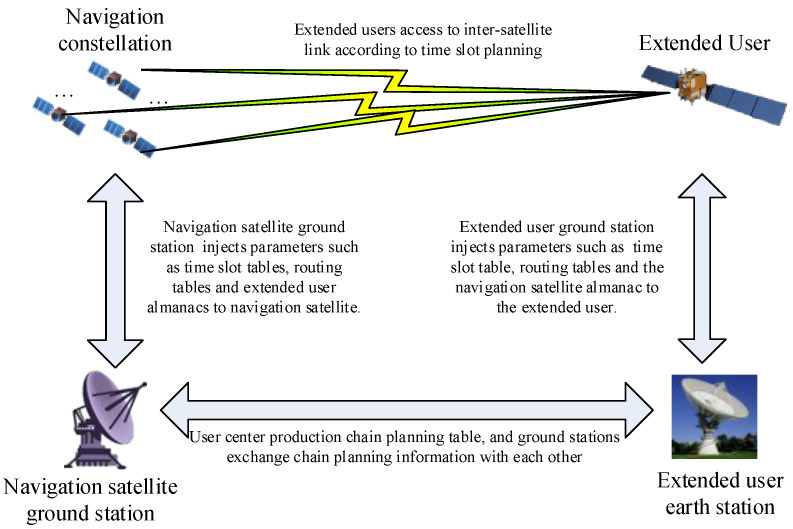
The process of an extended user accessing the navigation Ka-band inter-satellite link.

The Chinese navigation satellite system consists of 30 satellites, including geostationary orbit (GEO), inclined geosynchronous orbit (IGSO) and medium earth orbit (MEO) satellites. It has established a navigation inter-satellite link system capable of precise measurement and data transmission [12,13]. The process by which extended users access this system involves several stages. First, extended users must submit an access request based on their application requirements. Next, a visibility analysis is conducted according to the beam scanning ranges of both parties to plan the link establishment time slots, the number of links and the allocation of time slot resources in advance. Finally, when the conditions for link establishment are met, both parties use ephemeris data to obtain each other’s position and velocity information, completing spatial coordinate alignment to establish the link [14,15,16,17]. The process of extended user access to inter-satellite links includes three stages: link establishment preparation, unidirectional signal reception and bidirectional link establishment with the satellite. Extended users periodically submit usage requests, prompting the navigation satellite system to generate various operational parameters and provide them to the extended users. Daily data exchanges occur between the navigation satellites and extended users, including ephemeris, clock bias parameters and broadcast ephemeris data, enabling inter-satellite link access, as illustrated in Figure 1.

Accessing the navigation inter-satellite link requires stringent conditions, as extended users must meet specific time and frequency constraints, achieving time–frequency synchronization at the microsecond and hundred-hertz levels. Navigation satellites are equipped with high-precision atomic clocks, providing accurate time and frequency references for the entire satellite and enabling rapid bidirectional communication link establishment. Due to weight and power consumption constraints, extended users cannot be equipped with atomic clocks, resulting in time–frequency accuracy two to three orders of magnitude lower than that of navigation satellites. Given the limited time slot resources of the inter-satellite link, the extended user leverages its wide-range acquisition capability to capture unidirectional Ka-band signals. It then uses a small number of unidirectional satellite measurements to build a clock bias model, estimate clock bias parameters and apply compensation through time and frequency correction models, achieving high-precision time–frequency synchronization and enabling bidirectional communication.

**Figure 2:**
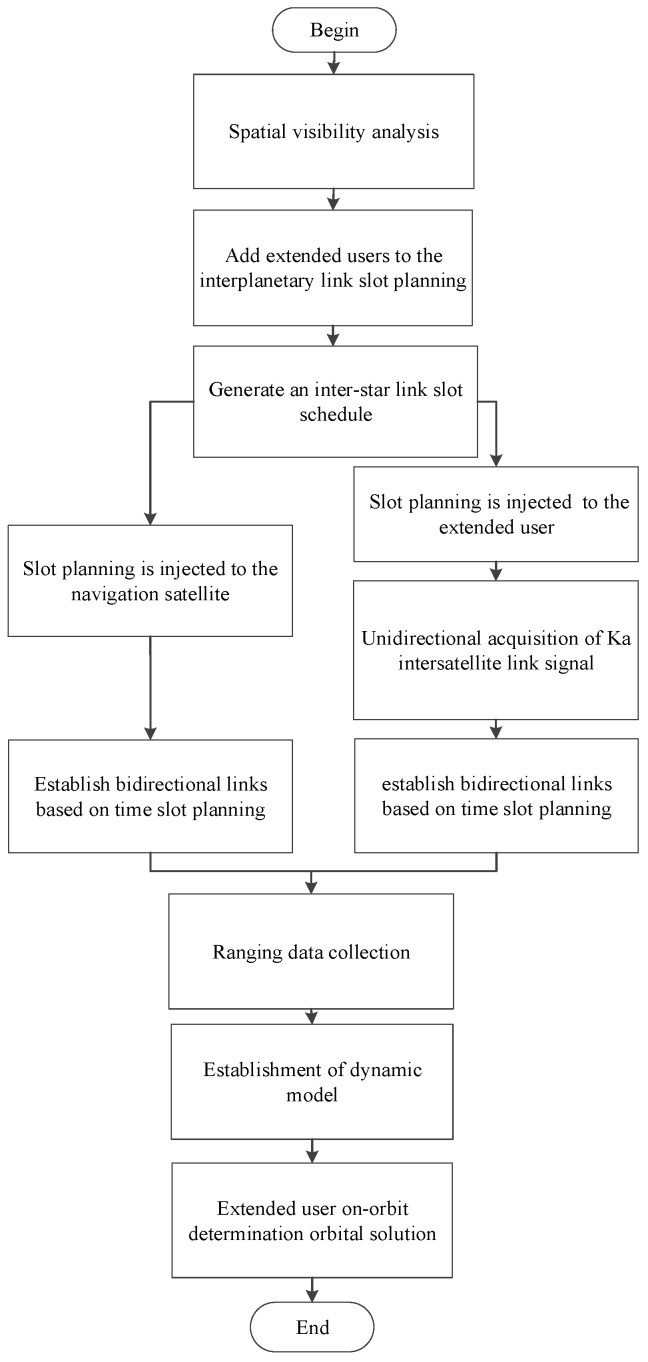
Orbit-determination process of extended users using the Ka-band inter-satellite link.

Based on bidirectional communication, there are two methods for orbit determination: (1) The extended user collects unidirectional ranging values while in orbit and performs real-time unidirectional orbit determination. This involves acquiring 3–4 inter-satellite link time slots per minute and collecting unidirectional ranging values for one hour per day over two days, developing a dynamic model and state transition matrix for orbit determination. (2) Bidirectional ranging values between the navigation satellite and the extended user are downlinked to ground stations via respective satellite–ground links, enabling post-processing orbit determination on the ground. To ensure timely orbit determination, this study primarily focuses on the research of real-time unidirectional orbit-determination technology for extended users in orbit, achieving an accuracy better than 100 m. The process of extended user access for unidirectional orbit determination is illustrated as follows in Figure 2 [8].

## 3. Time–Frequency Compensation and Measurement Model

### 3.1. Clock Model

Under the constraints of weight and power consumption, the extender cannot be equipped with an atomic clock with high time–frequency accuracy and only a high-stability crystal oscillator can be used. The original high-stability crystal oscillator output reference frequency is 10 MHz. The frequency accuracy of each measurement is calculated using the following formula:

(1) $$\Delta f_{r}=\frac{f-f_{0}}{f_{0}}.$$

Here, $\Delta f_{r0}$ is the frequency accuracy, *f* is the frequency-measurement value, and $f_{0}$ is the reference frequency.

After continuous measurement for two hours using a high-temperature crystal oscillator, the clock was in a stable state with a linear trend in frequency accuracy, and there was also a jitter change. By using linear fitting to analyze frequency accuracy data, frequency drift values can be obtained and fitting residuals can be given. Based on the above analysis, the clock frequency variation can be described using the following equation.

(2) $$\Delta f_{r}=\Delta {f_{r}}_{0}+Kt+\delta f_{r}.$$

Here, $\Delta {f_{r}}_{0}$ is the initial value of the frequency accuracy, *K* is the frequency drift rate and $\delta f_{r}$ is a random jitter that conforms to a normal distribution.

The relationship between clock uncertainty and clock frequency accuracy can be represented by the following equation [18,19,20]:

(3) $$\left\{\begin{array}{c} \delta t=m_{0}+m_{1}t+m_{2}t^{2}+\xi t,\\ m_{1}=\Delta {f_{r}}_{0},\\ m_{2}=\frac{1}{2}K,\\ \xi t=\int_{t_{0}}^{t}\delta f_{r}dt. \end{array}\right.$$

Here, δt is the clock bias at time *t*, $m_{0}$ is the clock bias at time $t_{0}$, $m_{1}$ is the clock speed term, $m_{2}$ is the clock drift term and ξt is the clock bias jitter at time *t*.

The observation model for the pseudorange observation value of navigation satellite *s* is as follows [21,22,23]:

(4) $$p_{f}^{s}=r^{s}+\delta t-\delta t^{s}-\Delta_{rela}^{s}+\sigma^{u}+\sigma^{s}+\varepsilon_{p_{f}}.$$

In Equation (4), $p_{f}^{s}$ represents the pseudorange observation value at frequency *f*; $r^{s}$ represents the geometric distance from the Ka antenna phase center of the Beidou satellite to the extended user’s Ka antenna phase center; δt and $\delta t^{s}$, respectively, represent the true clock bias of the extended user and the navigation satellite; $\Delta_{rela}^{s}$ represents the relativistic effect correction error caused by the relativistic delay, which can be ignored after correction; $\sigma^{u}$ and $\sigma^{s}$, respectively, represent the channel delay for extended users and navigation satellites; $\varepsilon_{p_{f}}$ represents the pseudorange observation noise at frequency *f*, whose initial values are pre-set.

The clock bias can be expressed as follows:

(5) $$\delta t=p_{f}^{s}-\left(r^{s}-\delta t^{s}-\Delta_{rela}^{s}+\sigma^{u}+\sigma^{s}+\varepsilon_{p_{f}}\right).$$

Neglecting the random jitter ξt caused by clock error, we construct the system of equations:

(6) $$\left\{\begin{array}{c} \delta t_{1}=m_{0}+m_{1}\Delta t_{1}+m_{2}\Delta t_{1}^{2},\\ \ldots \\ \delta t_{i}=m_{0}+m_{1}\Delta t_{i}+m_{2}\Delta t_{i}^{2},\\ \ldots \\ \delta t_{n}=m_{0}+m_{1}\Delta t_{n}+m_{2}\Delta t_{n}^{2}. \end{array}\right.\left(i=2,\ldots ,n-1\right)$$

According to the least squares algorithm, the clock difference parameters $m_{0}$, $m_{1}$ and $m_{2}$ can be fitted by the linear model. The time capture capability of the extended user equipment is at the millisecond (ms) scale, and the frequency capture capability is at the kilohertz (kHz) scale. After capturing the unidirectional signal, the clock bias $m_{0}$, clock velocity term $m_{1}$ and clock drift term $m_{2}$ calculated from the group of unidirectional measurements are used to adjust the time–frequency in orbit, which can achieve microsecond- and hundred-hertz-level time–frequency synchronization requirements and meet the conditions for bidirectional link building with the navigation constellation.

### 3.2. Unidirectional Orbit-Determination Motion and Observation Equation

**Figure 3:**
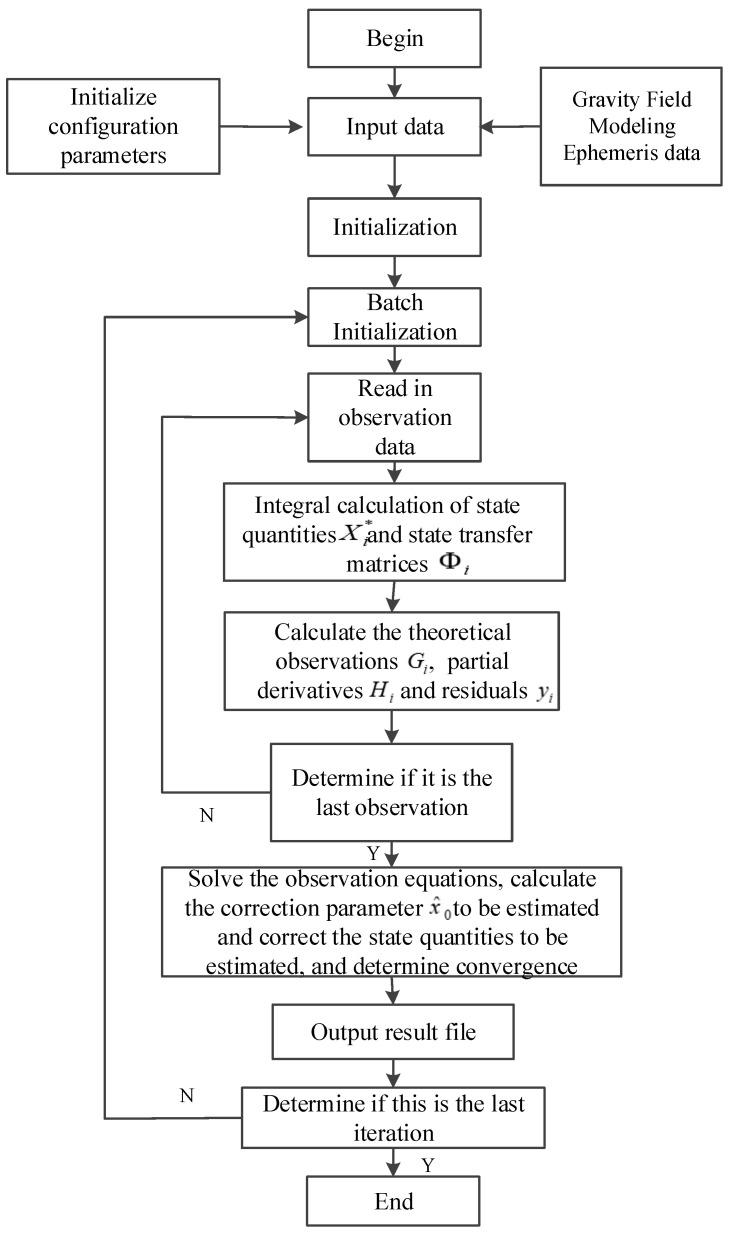
Extended user unidirectional orbit-determination algorithm flow.

After the extended user completes link establishment using a limited number of inter-satellite link time slots, the unidirectional measurements are initialized and processed in batch, state and observation equations are defined, and the parameters to be estimated are calculated and corrected. The process iterates until convergence criteria are met, enabling the determination of satellite orbit and clock bias parameters. The algorithm flow is shown in Figure 3 [24,25,26].

The geocentric inertial coordinate system is an inertial coordinate system that remains stationary in inertial space. The origin of the coordinate system is the center of the Earth; the fundamental plane is the equatorial plane of the Earth; the Z-axis is the axis of rotation perpendicular to the equatorial plane, pointing to the north pole of the Earth; the X-axis points to the vernal equinox; the Y-axis is perpendicular to the X-axis; and X, Y and Z follow the right-hand spiral rule. According to Kepler’s first law, the mass of a satellite can be considered negligible compared to the mass of the Earth. Assuming that the Earth is a uniform sphere with an orbital eccentricity of 0, the acceleration of a satellite can be given by Newton’s law of universal gravitation. The motion equation of the satellite in the Earth-centered inertial (ECI) frame is as follows [27,28,29]:

(7) $$\left\{\begin{array}{c} \dot{\vec{R}}=\vec{V},\\ \dot{\vec{V}}=-\frac{GM_{⊕}}{R^{2}}\frac{\vec{R}}{R}+\vec{P}\left(\vec{R},\vec{V},{\vec{P}}_{d},t\right)\equiv \vec{A},\\ \vec{R}\left(t_{0}\right)={\vec{R}}_{0},\dot{\vec{R}}\left(t_{0}\right)=\vec{V}\left(t_{0}\right). \end{array}\right.$$

The Equation (7) is as above, where $\vec{R}$ denotes the satellite’s center-of-mass position vector; $\vec{V}$ denotes the satellite’s center-of-mass velocity vector; $\vec{P}$ denotes the perturbing acceleration acting on the satellite; $\vec{A}$ denotes the total acceleration acting on the satellite; ${\vec{P}}_{d}$ denotes the vector of dynamics parameters to be estimated, which may include the solar radiation pressure coefficient, etc.; and $GM_{⊕}$ denotes the Earth’s gravitational constant.

Parameters that do not appear in the satellite’s motion equation are referred to as geometric parameters, denoted as $\vec{P}$. Definition of the state vector:

(8) $$X={\left[\begin{array}{cccc} {\vec{R}}^{T} & \vdots {\vec{V}}^{T} & \vdots {\vec{P}}_{d}^{T} & \vdots {\vec{P}}_{g}^{T} \end{array}\right]}^{T}.$$

At the time of observation *t*, the state variable *X* can be expressed as a differential equation with respect to time. The motion Equation (7) can be rewritten as the state equation:

(9) $$\left\{\begin{array}{c} \dot{X}=F\left(X,t\right)=\left[\overset{⇀}{\;V}\vdots \overset{⇀}{\;A}\vdots 0\right],\\ X\left(t_{0}\right)=X_{0}. \end{array}\right.$$

This is the dynamic equation of the satellite, which is established based on Newton’s second law, where *V* is the velocity vector of the satellite’s center of mass; *A* is the total acceleration experienced by the satellite.

The actual state vector *X* of the satellite cannot be known precisely in advance and can only be obtained as a reference value $X^{*}$. It needs to be continuously optimized using the constraints from the measurements, making it as close as possible to the true value. The observation quantity for unidirectional ranging is the distance $\rho_{0}$ from the satellite to the BeiDou satellite, and its calculated value is as follows:

(10) $$\rho_{C}=\sqrt{{\left(\overset{⇀}{\;r}-{\overset{⇀}{\;r}}^{sta}\right)}^{T}\left(\overset{⇀}{\;r}-{\overset{⇀}{\;r}}^{sta}\right)}.$$

In Equation (10), $\overset{⇀}{\;r}$ and ${\overset{⇀}{\;r}}^{\mathrm{sta}}$ represent the position vectors of the BeiDou satellite and the satellite in the Earth-fixed coordinate system, respectively. 

Usually, inter-satellite measurements use bidirectional measurements, and subtracting the bidirectional pseudorange measurements at the same time can eliminate satellite orbit information and leave only satellite clock error information. The sum of bidirectional pseudorange measurements obtained simultaneously can eliminate satellite clocks. This study proposes an observation equation based on unidirectional measurement and then performs error correction on the observed values, and linearizes and constructs the observation equation. After estimating the parameters of the observation equation, the initial state of the satellite and the satellite clock error parameters are obtained, and the optimal estimation of the parameters is obtained through residual editing and iteration.

Let $Y=\rho_{0}$ and $G\left(X,t\right)=\rho_{C}$; then, the observation equation is as follows:

(11) $$Y_{i}=G\left(X_{i},t_{i}\right)+\varepsilon_{i},\;i=1,\cdots ,n.$$

In Equation (11), $X_{i}$, $Y_{i}$ and $\varepsilon_{i}$ represent the satellite state vector, observation value and observation noise at time $t_{i}$, respectively.

The state Equation (9) and the observation Equation (11) represent the two fundamental relationships for determining the trajectory. Assuming that the reference state $X^{*}$ is sufficiently close to the actual trajectory, Equations (7) and (9) can be expanded at $X^{*}$ using a Taylor series, neglecting second-order and higher terms, resulting in the linearized fundamental relationships as follows:

(12) $$\left\{\begin{array}{c} \dot{x}=\dot{X}\left(t\right)-{\dot{X}}^{*}\left(t\right)=B\left(t\right)x,\;x\left(t_{0}\right)=x_{0},\\ y_{i}=Y_{i}-Y\left(X_{i}^{*},t_{i}\right)={\tilde{H}}_{i}x+\varepsilon_{i},i=1,\cdots ,n. \end{array}\right.$$

In Equation (12), combining the state equation and observation equation yields the following:

(13) $$\left\{\begin{array}{c} B\left(t\right)={\left.\frac{\partial F}{\partial X}\right|}_{X^{*}},\\ {\tilde{H}}_{i}\left(t\right)={\left.\frac{\partial G}{\partial X}\right|}_{X_{i}^{*}}. \end{array}\right.$$

According to the numerical integration method, the solution to the first equation in (12) is as follows:

(14) $$x\left(t\right)=\Phi \left(t,t_{0}\right)x_{0}.$$

In this Equation (14), $\Phi \left(t,t_{0}\right)$ is the state transition matrix, which is the solution to the following system of matrix differential equations:

(15) $$\left\{\begin{array}{c} \dot{\Phi }\left(t,t_{0}\right)=B\left(t\right)\cdot \Phi \left(t,t_{0}\right),\\ \Phi \left(t_{0},t_{0}\right)=I. \end{array}\right.$$

The state variable *X* is propagated from time $t_{0}$ to time *t* using the state transition matrix.

Substituting Equation (14) into the second equation of (12) yields the linearized observation equation:

(16) $$y=Hx_{0}+{\bar{P}}_{0}.$$

In Equation (16), ε is the observation noise and ${\bar{P}}_{0}$ is the covariance moment:

(17) $$y=\left(\begin{array}{c} y_{1}\\ \vdots \\ y_{n} \end{array}\right),\;H=\left(\begin{array}{c} {\tilde{H}}_{1}\Phi \left(t_{1},t_{0}\right)\\ \vdots \\ {\tilde{H}}_{n}\Phi \left(t_{n},t_{0}\right) \end{array}\right),\;{\bar{P}}_{0}=\left(\begin{array}{c} \varepsilon_{1}\\ \vdots \\ \varepsilon_{n} \end{array}\right).$$

The best estimate of $x_{0}$ can be obtained from Equation (16) using the least squares method:

(18) $${\hat{x}}_{0}={\left(H^{T}WH\right)}^{-1}H^{T}Wy.$$

In this Equation (18), *W* is the weight matrix of the observed data.

Given the prior estimated parameter ${\bar{x}}_{0}$ and the prior covariance matrix ${\bar{P}}_{0}$, the estimate at time *t* is as follows:

(19) $$\hat{x}={\left(H^{T}WH+{\bar{P}}_{0}^{-1}\right)}^{-1}\left(H^{T}Wy+{\bar{P}}_{0}^{-1}{\bar{x}}_{0}\right).$$

The improved trajectory is then obtained [30]:

(20) $$\hat{X}=X^{*}+\hat{x}.$$

The above estimation is obtained based on linearization, which requires the initial value to be close to the true value; however, this is difficult to ensure in practice. Therefore, an iterative method is required to repeatedly solve the process, with the most recent estimate used as the reference value for linearization in each iteration, continuing until the convergence criterion is met.

## 4. Orbit-Tracking and -Determination Algorithm Simulation

### 4.1. Accuracy of Clock Difference Calculations

**Figure 4:**
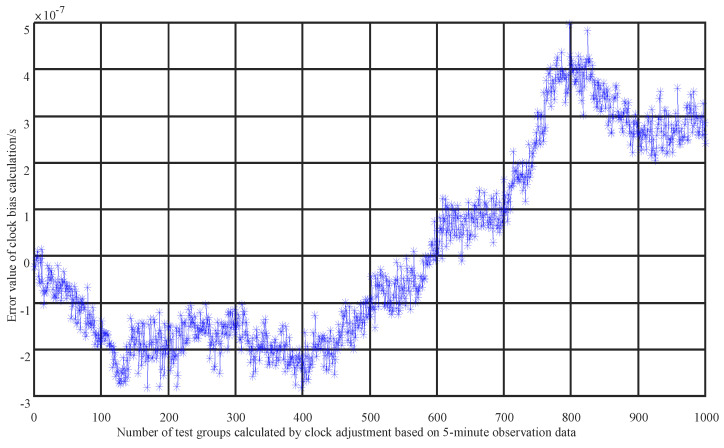
Clock offset solution accuracy of 20 sets of unidirectional range measurements (* and blue line represent the error value of clock bias calculation).

**Figure 5:**
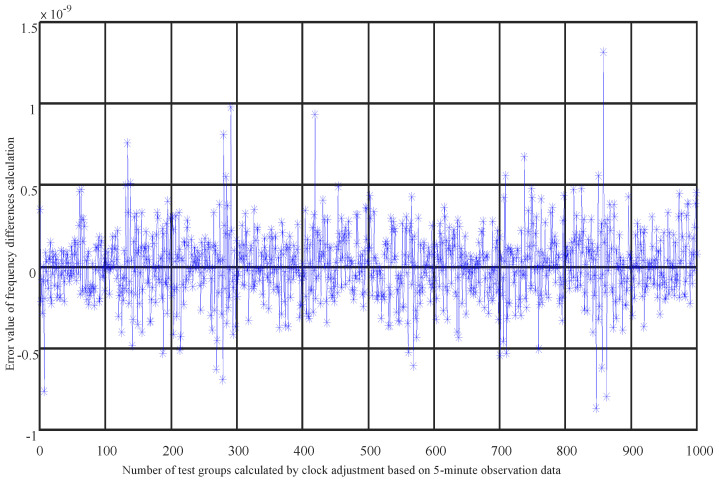
Frequency offset solution accuracy of 20 sets of unidirectional range measurements (* and blue line represent the error value of frequency differences calculation).

Extended users, constrained by weight and power consumption, cannot be equipped with high-precision atomic clocks. The initial error of the clock offset parameter a0 can reach 5 ms, and that of a1 can reach 5 kHz, with the time–frequency accuracy being two to three orders of magnitude worse than that of navigation satellites. These extended users rely on their own wide-range capture capabilities to establish unidirectional communication links with navigation satellites, connecting with different satellites each minute. We select the latest 20 sets of unidirectional test data from the past 5 min for preprocessing, then correct the observational values, linearize them and form observation equations. After estimating the parameters of the observation equations, we obtain the initial state of the satellites and the satellite clock offset parameters. The optimal estimation of these parameters is achieved through residual calculation and iteration, as shown in Figure 4 and Figure 5. It can be seen that the solution accuracy for a0 is better than $5\times 10^{-7}$ seconds, equivalent to 0.5 microseconds (us), and the solution accuracy for a1 is better than $1.35\times 10^{-9}$, equivalent to 31 hertz (Hz), meeting the time–frequency synchronization requirements at the microsecond and tens-of-hertz levels. The time accuracy has been improved by three orders of magnitude, and the frequency accuracy by two orders of magnitude. This method solves the problem of non-atomic clock users achieving high-precision time–frequency synchronization with fewer time slot resources and navigation satellite links, providing a new solution for time–frequency synchronization for other users.

### 4.2. Simulation Results of Orbit-Determination Precision

**Figure 6:**
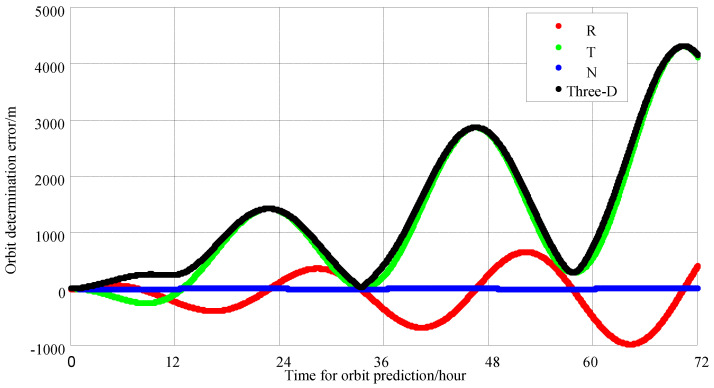
One-hour daily observation with 8 time slots per minute, for a 1 day period; the 72 h orbit error chart.

Due to the fact that the navigation satellite links conform to the Walker constellation and are evenly distributed globally, the specific timing of expanding user access does not affect the accuracy of orbit determination. The main errors in orbit determination are caused by clock accuracy error, antenna phase center deviation, channel delay error, observation noise, relativistic effects and other errors. After these errors are set to predetermined values, we mainly analyze the impact of the number of time slots and access time per minute on the accuracy of orbit determination. Therefore, we only analyze the duration of access and the number of navigation satellites connected each time. The more satellites the extended user accesses with the navigation satellite and the longer the observation arcs are, the higher the measurement precision is. However, the time slot resources for navigation satellite links are limited. Increasing the number of measurement satellites and arcs can burden the navigation satellite links themselves, affecting their normal functions. Therefore, it is necessary to analyze the impact of observation arcs and time slots on orbit-determination precision. The simulation scenario is set for GEO orbit extended users, with initial orbital errors and clock offsets set at 10 km and 5 ms, respectively. The navigation satellites with the best position dilution of precision (PDOP) values are selected for building the link in each link establishment cycle. According to the internal operation strategy of the constellation, a maximum of eight time slot resources per minute are allowed for extended users to use without affecting its own operation. Observations are made for 1 h per day, with three, four and eight link building time slots per minute. Orbit determination and forecasting are conducted using 2 days and 1 day of observation data, respectively, for 24 h and 70 h forecasts, and the maximum three-axis errors are statistically forecasted for 24 h. Orbit determination is performed with 1 h of daily observation and eight time slots per minute, using 1 day of observation data. The results of the orbit-determination simulation are shown in Figure 6. Here, R represents the radial component, T represents the component along the motion trajectory direction, N represents the normal component perpendicular to R and T, and Three-D represents three-dimensional component, respectively. The simulation indicates that the orbit error for a 24 h forecast is 1500 m, and the orbit error for a 72 h forecast is more than 5000 m.

**Figure 7:**
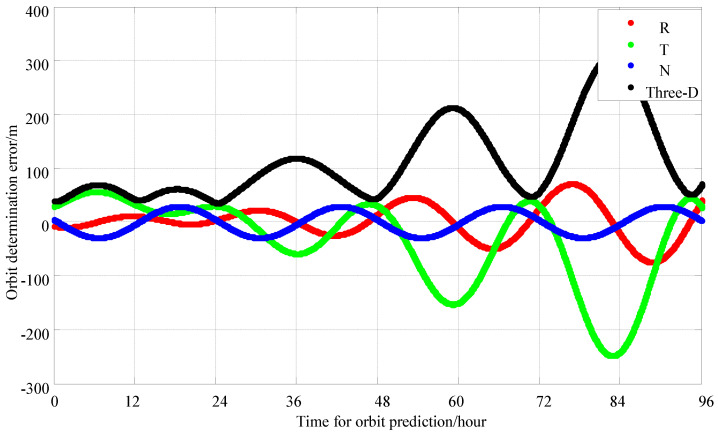
Data for 1 h daily observations, a 2 day period and 4 time slots per minute; 72 h orbit error-prediction chart.

Observations are made for 1 h each day, with four time slots per minute, using data from a 2 day observation period, and calculations are performed every hour with a sliding window, as shown in Figure 7. The simulation results indicate that the predicted 24 h orbit error is 80 m, and the predicted 72 h orbit error is 205 m.

**Figure 8:**
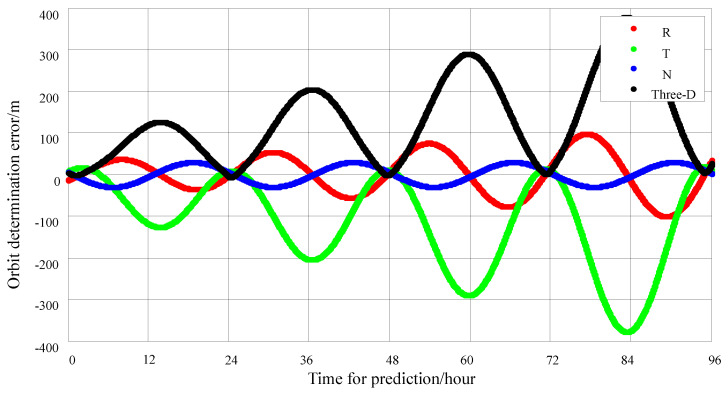
Data for 1 h daily observation, a 2 day period and 3 time slots per minute; 72 h orbit error-prediction chart.

Observations are made for 1 h each day, with 3 time slots per minute, using data from a 2 day observation period, and calculations are performed every hour with a sliding window, as shown in Figure 8. The simulation results indicate that the predicted 24 h orbit error is 140 m, and the predicted 72 h orbit error is nearly 300 m.

We have compiled the simulation results under different conditions, as shown in Table 1. The extended users utilize inter-satellite links to collect unidirectional ranging values, employing a unidirectional orbit-determination strategy. Observations are made for 1 h each day, with four time slots per minute, over a 2 day observation period. The accumulated observation data are updated on a rolling basis, achieving a position forecast accuracy of 80 m within 24 h, which is on par with GNSS receivers and represents a leading level domestically.

### 4.3. In-Orbit Measurement Results for Orbit Determination

**Figure 9:**
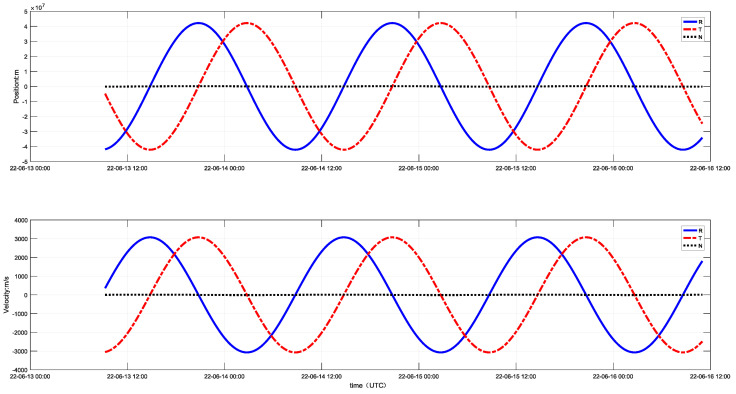
Extended user’s in-orbit orbit-determination results.

To better verify the unidirectional orbit-determination algorithm, the extended user conducted in-orbit validation. After the extended user was launched into orbit, an application for access to the inter-satellite link was made and planning for the link arc segment and time slot resources was carried out in advance. Both parties obtained each other’s position and velocity information from the almanac, thereby obtaining the spatial coordinate pointing. Once the extended user entered the link mode, it utilized a wide-range capture capability to complete unidirectional link establishment and selected 5 min of 20 sets of unidirectional ranging data for clock adjustment and calculation. Under the condition of meeting the inter-satellite link’s time and frequency precision constraints, bidirectional link communication was achieved. Based on the telemetry data received on the ground, measurements from universal time coordinated (UTC) 13 June 2022, to 16 June 2022, were selected. The extended user accessed the inter-satellite link for 1 h each day, establishing links with four different navigation satellites every minute within that hour. A 2 day sliding window of ranging values was used for orbit-determination calculation, with the results shown in Figure 9. Analysis indicates that the inter-satellite link’s orbit-determination performance is stable, with a 100% availability rate for the determined orbit values and ranging precision better than 0.6 m. Here, R represents the radial component, T represents the component along the motion trajectory direction and N represents the normal component perpendicular to R and T.

**Figure 10:**
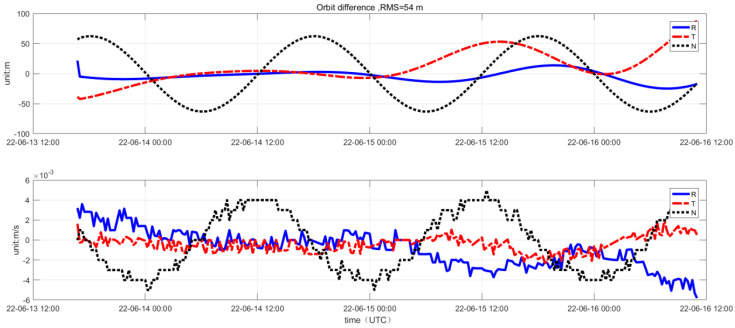
Comparison of orbit-determination results with GNSS orbit-determination results.

To further analyze the precision of the in-orbit orbit-determination results, a comparative analysis of the in-orbit orbit-determination accuracy was conducted using the satellite platform GNSS orbit-determination results and the ground orbit-determination results as benchmarks. The in-orbit orbit-determination results were compared by calculating the differences in velocity and position values with the control and satellite platform GNSS orbit-determination results. The precision analysis results are shown in Figure 10. Data from 13 June, after the GNSS orbit determination stabilized, were selected, showing an orbit-determination position error of 54 m and a velocity error of 0.035 $m/s^{2}$.

**Figure 11:**
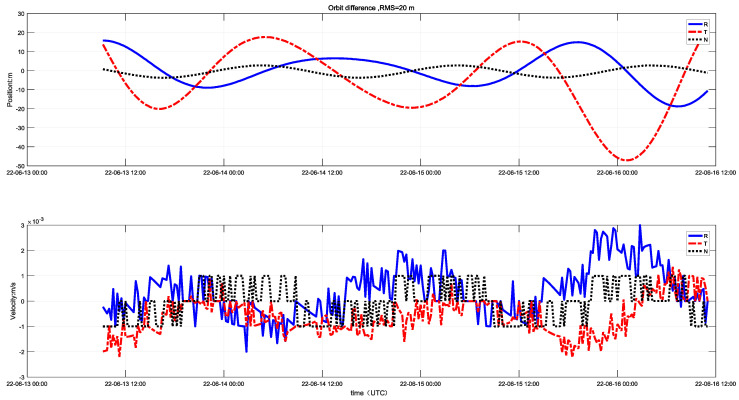
Comparison of orbit-determination results with the orbit determination from the ground station.

By comparing the in-orbit orbit-determination results with the high-precision orbit-determination velocity and position values from the ground station, a precision analysis was conducted. The results are shown in Figure 11. The orbit-determination position error is within 20 m, and the velocity error is $3\times 10^{-3}\;m/s^{2}$. From the in-orbit measurement data, it can be seen that, with four time slots per minute and one hour of observation per day, the unidirectional orbit-determination results from a 2 day test are within 100 m of the GNSS results. Compared to the more precise ground high-precision orbit determination, the orbit-determination error is within 20 m. The results are on the same level as those of GNSS orbit determination and can surpass them under good error control conditions.

## 5. Conclusions

In this paper, a unidirectional orbit-determination method for non-atomic clock extended users using navigation Ka-band inter-satellite links is innovatively proposed. After the extended user completes angle pointing calculation and unidirectional capture tracking, under the condition of limited navigation inter-satellite link time slot resources, it collects unidirectional ranging values between the extended user and navigation satellites per minute. The clock offset model is used to calculate and compensate for the clock offset, improving the time precision by three orders of magnitude and the frequency precision by two orders of magnitude, achieving high-precision time–frequency synchronization and bidirectional communication. After the establishment of the bidirectional link, with three to four inter-satellite link time slots per minute and one hour of observation per day, two days of unidirectional ranging values are collected to establish a mechanical model and state transition matrix for real-time orbit determination and calculation.

For the first time in China, this technology has enabled extended users to access navigation inter-satellite links and achieve hundred-meter-level orbit determination. Compared with traditional space–ground control links, it can provide 24 h timing for spacecraft without geographical restrictions and, compared with conventional methods of accessing navigation inter-satellite links, it occupies fewer navigation inter-satellite link resources and does not affect the normal functions of the navigation constellation. This technology solves the problem of overseas extended users utilizing limited time slot resources for emergency measurement and orbit determination, greatly enhancing the robustness of extended users. It provides strong technical support for various applications of navigation Ka-band inter-satellite links in China.

## Figures and Tables

**Table 1:** Unidirectional orbit-determination result analysis.

Scheme	Data Arc Segment	Time Slots per Minute	Time Slots Within 1 h	Observation Period	24 h Three-Axis Synthetic Error (m)	72 h Three-Axis Synthetic Error (m)
1	1 h each day	8	480	1 day data processing	1500 m	5000 m
2	1 h each day	4	240	2 day data processing	80 m	205 m
3	1 h each day	3	180	2 day data processing	140 m	300 m

## Data Availability

Data are contained within the article.

## Abbreviations

The following abbreviations are used in this manuscript:

GNSS	Global Navigation Satellite System
TT&C	Tracking, Telemetry and Command
GPS	Global Positioning System
GEO	GEostationary Orbit
IGSO	Inclined GeoSynchronous Orbit
MEO	Medium Earth Orbit
ECI	Earth-Centered Inertial
ms	milliseconds
kHz	kilohertz
us	microseconds
PDOP	Position Dilution of Precision
UTC	Universal Time Coordinated
